# Centering caregiver voices on caregiving needs of children with neurodevelopmental disorders in low-resource settings

**DOI:** 10.1371/journal.pone.0335402

**Published:** 2025-10-30

**Authors:** Tamara Chansa-Kabali, Martha Banda-Chalwe, Joseph Lupenga

**Affiliations:** 1 Department of Psychology, School of Humanities and Social Sciences, University of Zambia, Lusaka, Zambia; 2 Centre for Research in Disability, Rehabilitation and Policy Development, Lusaka, Zambia; 3 Department of Epidemiology and Biostatistics, School of Public Health, University of Zambia, Lusaka, Zambia; Christian Medical College, INDIA

## Abstract

Caregivers of children with neurodevelopmental disorders (NDDs) in low-resource settings face significant challenges in accessing appropriate care, support, and interventions. This study was centred on caregivers’ lived experiences as a channel through which research and intervention planning draws a better understanding of experiences, barriers, and needs for providing care. Using qualitative methods, we applied the interpretive phenomenological approach to navigate caregiving needs required to optimise the caregiving experience. Findings reveal that caregivers navigate multiple hardships, including limited knowledge and care skills on NDDs, access to rehabilitation services, and heightened social stigma exemplified through lack of empathy in families and communities. Despite these challenges, caregivers demonstrate remarkable resilience and develop adaptive strategies to support their children’s development by identifying ways that empower caregiving—increased knowledge, use of rehabilitation services in community spaces, empathy and thriving together through support groups. By centering on caregiver perspectives, this research informs more inclusive and sustainable approaches to supporting families of children with NDDs in resource-constrained environments.

## Introduction

Disability is a lived phenomenon in all parts of the world. Global reports show that at least fifteen percent, or one billion of the world’s population have a disability [1]. Of these, 240 million are children [2], with the majority living in the global south [3] and over 53 million clustered as developmental disabilities [4,5], among which are neurodevelopmental disorders (NDDs) [6]. In this paper, we operationalise NDDs in accordance with the diagnostic and statistical manual of mental disorders (DSM) fifth edition [6] as a diverse set of lifelong conditions (i.e., intellectual disabilities [ID], autism spectrum disorders [ASD], communication disorders [CD], attention deficit hyperactivity disorders [ADHD], specific learning disorders, motor disorders, cerebral palsy, and epilepsy among others) with propensity for increased comorbidities [7]. Further, these NDDs are underlying conditions arising from impairments in the developing brain and central nervous system causing a decline in cognitive functioning, delays in language skills, poor motor, adaptive and social-emotional skills [8] and may require continuous caregiver assistance.

Records show that NDDs are higher in low- and middle-income countries (LMICs) due to poor pre-, peri and postnatal conditions [9,10]. These higher rates are exacerbated by 1) improved child survival rates, growing at-risk infant population [11–16], 2) persistent parental risk factors—poverty, education, literacy levels, marital status, maternal BMI, physical and mental health, age, substance abuse and exposure to chemicals [17–22] and 3) inadequately treated perinatal conditions (i.e., preterm, low birthweight, low Apgar scores, oxygen deprivation, parasitic infections, early brain injury and nutritional deficiencies) [21,23,24]. Zambia, a poorly resourced country, experiences increased risk for prenatal complications [10,23,25], but has no actual prevalence data due to the unavailability of national disaggregated data on NDDs. However, estimates from the 2015 National Disability Survey show 4.4% disability prevalence among children aged 2–17 years, of which approximately 90% are NDDs [26] giving significant estimates for planning public health interventions and research activities.

Research converges on the urgent need for early intervention in children with NDDs [8,27]. This call is anchored on the evidence that children with NDDs when compared to children without are 34% more likely to experience stunting, 42% less likely to possess foundational literacy and numeracy skills, 24% less likely to receive responsive care and early stimulation, 25% less likely to attend early childhood education [21,28–30]. Access to early intervention resources for NDDs is especially essential for families to navigate the complex clinical and educational needs in poor contexts where many families face neglect in social and health provisions [31]. Without formal institutional supports for children with NDDs, care is inevitably the responsibility of the immediate family and, in many cases, the biological mother or maternal grandmother [32–36]. The general and specific poor context of care for children with NDDs heightens the burden, leaving caregivers overwhelmed, stressed, depressed, anxious and discouraged [31,37–42]. Despite the caregiving difficulties, caregivers show resilience and plasticity [43] in caring for children with NDDs [44,45].

In the recent past, there has been growing recognition of positive outcomes for both children and their families utilising family-centred interventions embedded in community-based approaches [20,46–48]. This evidence shows the influence of family care practices in reducing detrimental effects of social disadvantage on brain structure and function [45], strengthening the ideology that local people possess the assets to handle their problems [32,44,48,49]. Caregivers’ role in rehabilitative and educational interventions sets them apart in parent-guided interventions, emphasising mutual respect and co-creation of meaning [21,43,50–52]. Therefore, exploring socio-cultural nuances of caregiving requirements to ensure sustainable care for children with NDDs in resource-constrained settings remains imperative. By focusing on caregivers, we acknowledge the family’s consistent and integral role in child development while providing unique insights for engaging and working with families. Thus, this study sought to identify caregiving affordances and opportunities to inform intervention planning and address factors that may considerably restrict early learning opportunities for children with NDDs.

## Materials and methods

### Study design

The qualitative research design using the interpretive phenomenological approach was applied to capture unique lived experiences of caregivers of children with NDDs. This approach allows for unearthing phenomena from the perspective of how people interpret and attribute meaning to experiences, which largely influence their existence [53–55]. It also enriches understanding of meaning, terms of existence through referential associations by way of uncovering and disclosing experiences that may not easily resurface [56], taking advantage of the common social reality as experienced by a group of people to construct meaningful totalities out of the scattered events [57]. To enhance the rigour of the design, we applied bracketing, recognising that the first author is a parent of a child with an NDD, while serving as a professional and a researcher in the field. Bracketing followed personal and transpersonal reflection and reflexivity [58], constantly discussing the research process (data collection and analysis) with the co-researcher (second-named author), a health care professional with extensive expertise in disability studies. This allowed for the process of thinking together, transcending personal boundaries and producing a single thinking process [59]. The insider view worked as a strength worth adopting, serving as a source of additional insights and clarifications in the research process, increasing and solidifying the scope of validity, yet remaining faithful to participants’ accounts.

### Study population and sampling strategy

The study population consisted of the primary caregivers of children with NDDs in three districts—Lusaka, Kafue and Chongwe in Lusaka Province of Zambia. We operationalised primary caregiver as the person responsible for the day-to-day decision-making and care of the child. The caregiver participants for this study were purposively recruited by trained habilitation workers from Cheshire Homes Society of Zambia (CHSZ) between 19^th^ and 27^th^ January, 2023. CHSZ is an organisation that uses community-based approaches in child habilitation of underprivileged poor communities. CHSZ implemented a programme initially referred to as Get to Know Cerebral Palsy (GTKCP), built on the evidence of the value of self-help groups to improve maternal and child health outcomes. This programme was later expanded to include other NDDs and was renamed as “Ubuntu: working together with families of children with developmental disabilities,” and can be accessed at (https://www.ubuntu-hub.org/). The programme enables caregivers to start an empowerment ‘journey’ as soon as possible after the child’s diagnosis. Through duo facilitated sessions, a health worker and a champion mother (caregiver of a child with an NDD) were trained in the programme to complement strengths in working with groups of caregivers (10 per group). The model uses a participatory approach, emphasising adult learning principles, and caregivers sharing experiences and reflections. This allowed caregivers to identify, prioritise problems and choose ways to solve these problems together, whileensuring hands-on practice with the children. The programme covers a total of 12 topics (i.e., know your child, positioning, eating and drinking, learning to move, communicating, play, everyday activities, togetherness and belonging, and our community) with a desired timeframe of one topic per week. Inclusion criteria considered primary caregivers of children with a confirmed NDD diagnosis, aged 0–14 years at the time of data collection.

Following the project design of one focus group discussion (FGD) per district, we also applied convenient sampling to recruit a total of twenty-five (25) caregivers (8–9 per group) to participate in a single session of FGD. These FGD groups were largely homogeneous in socio-economic status, primarily because CHSZ serves underserved poor communities. Participant distribution included eighteen mothers, five grandmothers and two fathers, giving rich accounts of a father’s experience as primary caregivers. Group variation observed, particularly in age and cultural backgrounds, enriched sessions with intergenerational perspectives of NDDs.

Table 1 shows the general caregiver and family characteristics. These demographics show generally lower socio-economic parameters following level of education, income and occupation (with only 3), obtaining consistent monthly income through employment.

**Table 1:** Caregiver and family demographics.

Characteristics			
**Caregiver**		**Family**	
Age in years, Mean (range)	39 (18-72)	Single parent	12
**Gender** Male (Female)	2 (23)	Two-parent	13
**Education level**		**Household Size**	
Some primary	12	2-3	3
Some secondary	13	4-6	17
**Employment**		7-9	2
Employed*	3	10+	3
Casual Business**	22	Income per month, Mean (range)	K700 (K0-3000) ~$26 (111)

* Employment: civil service, retail shops.

** Casual Business: covering several income-generating activities—crop and livestock farming, seasonal businesses, charcoal processing and selling.

Table 2 shows the child’s mean age of 8 years, ranging from 1 to 14 years. The majority of the children were male (19), and only a handful (7) from the Chongwe district attended school. The most reported disability was cerebral palsy (12), comorbidities of CP and epilepsy (9), CP and down syndrome (2), autism (3), hydrocephalus and intellectual disability (2) and spina bifida and intellectual disability (1). All children presented some level of functional difficulties, including mobility, partial visual and hearing, talking (receptive and expressive language), self-feeding, feeding, bathing and dressing that was mostly assisted.

**Table 2:** Child demographics.

Child Characteristics	Frequency
Age in years Mean (Range)	8(1-14)
Gender Male (Female)	19(10)
In school, Yes (No)	7(22)
CP*	12
CP + Epilepsy	9
Autism	3
Down syndrome + CP	2
Hydrocephalus + ID**	2
Spina Bifida + ID	1
**Functioning difficulties**	
Mobility	16
Vision	2
Hearing	3
Talking	18
Toileting	18
Self-feeding	15
Feeding	20
Bathing	29
Dressing	28

* Cerebral palsy

** Intellectual disability

### Data collection

We collected data using the FGD method. Each FGD commenced after obtaining written informed consent from all participants for participation, taking notes and audio-recording. FGD sessions were conducted from neutral places within the area where caregivers routinely gathered for activities with CHSZ (Lusaka—school/clinic combined facility, Chongwe—school and Kafue—clinic). All sessions were co-facilitated (TCK as Main Moderator and MBC as Assistant Moderator). Sessions were structured to uphold confidentiality, encourage participation through the use of a local language (Cinyanja) and required code-switching between English and Cinyanja for both facilitators and participants. We used predetermined short, open-ended, non-threatening questions to elicit responses reflecting caregivers’ experiences [53,60–62], followed by probes to navigate deeper insights. In addition to the audio recordings of FDG1, FDG2 and FDG3, which lasted 166, 162 and 158 minutes respectively, we took hand-written field notes, particularly capturing nonverbal interactions of the sessions. The audio records were transcribed into clearly typed wording, including all observed non-verbal expressions. We observed that data saturation was reached by the third interview session, as indicated by the recurrence of comparable thematic patterns in caregivers’ accounts.

### Rigour and trustworthiness

The researchers ensured the study information was accurate by safeguarding the rigour and trustworthiness through the inclusion of confirmability, credibility, dependability and transferability [54,61,62]. The study’s credibility was enhanced by methods that ensured authentic data and interpretation. Prolonged participant engagement-built rapport and trust, while peer debriefing with co-authors provided critical feedback on methodology and interpretations [63,64]. To enhance transferability, the study detailed the research context, participants, sampling strategy and study setting. Dependability was ensured through meticulous documentation, maintaining an audit trail of data collection, analysis, and decision-making processes, ensuring transparency and replicability [63,64]. A code-recode strategy was used to confirm interpretation consistency. Confirmability was achieved by grounding findings in data, avoiding researcher bias. The audit trail included raw data, such as interview transcripts and field notes, evidencing the methodology. Reflexivity was employed, prompting researchers to examine their subjectivity and its influence [63,64].

### Data analysis

Thematic analysis was utilised for the analysis. Through this method, we searched for repeated patterns, particularly experiences, thoughts and behaviours within the data [65,66]. Thematic analysis offers an opportunity to focus on participants’ meanings and experiences by gaining insights into the internal and external reality of caregiving experiences to support the development of knowledge about reality. Additionally, using thematic analysis accentuates the social, cultural, and structural contexts influencing experiences, facilitating knowledge as constructed through interactions between researchers and the research participants, revealing meanings that are socially constructed [67]. To analyse the data, the inductive approach was specifically applied to identify latent “themes” reflecting patterned responses and or meanings derived from the data [66] using the six-step process of 1) familiarisation of raw data; 2) generation of coding framework 3) identifying themes and patterns 4) mapping themes reviewed against the data 5) verification and validation of themes 6) extraction of participant quotes, narratives, reporting and data representation.

### Ethical considerations

The study obtained ethical approval from the research ethics committee at the University of Zambia, under the *IORG No. 0005376*, *HSSREC IRB No. 00006464* and approval reference No. HSSREC: 2022-DEC-015. All the principles of ethics in research were observed during the study, including the provision of information concerning the study and the obtaining of consent in a language well understood by caregivers before and during FGDs [54,62].

## Results

**Fig 1:**
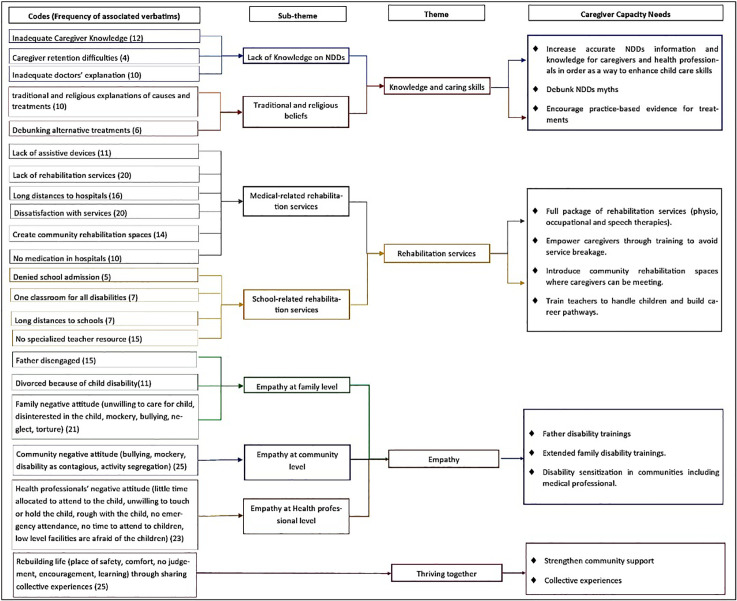
Thematic mapping of key themes and subthemes.

The data was manually analysed using the 6-step inductive iterative process proposed by Braun and Clarke [65]. Overall, the findings reveal four key themes for caregivers to apply in order to increase their capacity to care for children with NDDs. The derived themes as captured in Fig 1 below include: 1) knowledge and caring skills, 2) rehabilitation services, 3) empathy, and 4) thriving together. While a lack of finances is an unavoidable reality due to the rampant poverty situation, our analysis focused on uncovering caregiving ideas beyond finances.

### Theme 1: Knowledge and caring skills

Caregivers identified knowledge on NDDs as the foremost anchor for caregiving, leveraging understanding as the channel for child support and course of action. To caregivers, knowledge empowers action and is instrumental for care decisions. Notwithstanding the significance of NDDs knowledge on caring skills, we generally found limited NDDs knowledge among caregivers, with few knowing a little more than others. While nearly everyone could describe NDDs simply as a disability, only six of the twenty-five participants, one or two in each discussion group could clearly explain causes, name their child’s specific diagnosis, and detail both pharmacological and non-pharmacological care regimen. All caregivers reported to depend on clinicians for information, albeit their understanding remaining narrow. To the caregivers, confronting an NDD for the first time, required heavy reliance on professionals for both information and day-to-day guidance. However, many caregivers appeared to be discontent with the limited and “unsure” information that clinicians provided. Solemnly, caregivers shared, “*they are the doctors, they went to school, learned about these things and are qualified, that is why they are working*” (father, rural). Caregivers’ recollection of NDDs information provided by health professionals was more about differential patterns (slowness) of development that the child will experience than explanations of why. “*For me, I was only told that my child will not grow like other children, that’s all what the doctor said, I went out with a lot of questions*” (mother, urban). From the data, we observed that caregivers’ difficulties (e.g., illiteracy) in retaining and recalling information about NDDs could be a plausible explanation of the gap in knowledge. To this effect, caregivers relied on behavioural descriptors of the child’s struggles (e.g., does not sit, walk, talk, etc.). In the same vein, we also found that lack of knowledge, coupled with deeply rooted cultural beliefs, family/community advise and desperation for solutions, led caregivers to seek answers from traditional and religious healers. Many of these experiences entail practising “*scary*” rituals in the hope that the evil surrounding the child disappears, restoring the child to normality. One narrated, “*you cannot think straight, you just go with what people tell you because you have no grounding of what is going on, all you want is your child to be well”* (mother, urban). Another shared, … “*I was told to catch a live fly, kill it and mix with goat poop, and use the concoction on the incisions that I made on my son’s knees to enable him to walk*” (mother, rural).

It was clear from the results that the lack of knowledge affected caregiving. “*Maybe we do not know what to do, because for me, I have been following what we are taught by different organizations, but I do not see any change. My child is 13 years old now but he still cannot sit in one place, he even gets lost, maybe I just do not know how to help him, you can help me*” (mother, urban). Another said, “*we do not know what to do to help our children grow and do things independently, even sitting, walking, playing, or talking, these are challenges”* (grandmother, urban)*.* One caregiver, speaking with evident regret, said that because she lacked the necessary caregiving skills, her sole routine was to take her child outside each morning for a brief moment of sunlight before bringing the child back indoors*.* By contrast, another caregiver shared skill-enhancing activities despite the limited knowledge, “*when it’s morning, I take my child outside the house so that he can see different things. Then, where his friends are, I place him in a dish with a blanket inside so that he can be happy to watch his friends play. Then I also take a plate with stones and put the stones in front of him because he never used to be able to grip anything…. so, he would keep attempting to pick the stones and this child of mine, now manages to pick food from his plate to feed himself*” (mother, urban). Throughout the discussions, we observed caregiver readiness and commitment to adopt care systems that translate into developmental gains targeted at fostering child independence.

### Theme 2: Accessing rehabilitation services

The full utilisation of rehabilitative services was viewed as a way to enrich the caregiving experience. Caregivers appreciated the indispensable function of rehabilitation services, which, without doubt, was designed to ease the caregiving experience. From the narrations, caregivers who consistently used rehabilitation services reported child improvements. Despite the benefits of rehabilitation services, caregivers’ experiences centre on limited or non-existent services at local health facilities and community spaces. This is in spite of the children being in dire need of such services as physio, occupational and speech therapies. Other factors affecting access to rehabilitation services included constraints in personnel at facilities, specialised services, pharmacological options and long distances to facilities. Long distances with non-affordability of assistive devices such as wheelchairs hampered access to local and specialised health facilities. One caregiver said, *“my child has grown, he is 14 years old, I am not able to carry him on my back and walk to the clinic or hospital where my child is usually referred to. I have not been able to purchase a wheelchair to help me with movements, I cannot afford it” (mother, rural).* Long distances and a lack of finances subjected children to inconsistent access to medical attention and rehabilitation services, albeit higher vulnerability to illnesses. Additionally, caregivers reported longer waiting times at facilities that hindered service completion.

Similarly, caregiver experiences with educational services were appalling, “*schools refuse to accept our children on the premise that they do not have resource teachers trained in special education”* (mother, rural). Caregivers showed misgivings concerning schools’ capability and empathy to care for children, especially in the absence of self-care skills like toileting and classified children as developmentally “*young*” and “*not ready for school*”. “*I did not go back to the school, the teacher can just be troubling my child, so it is better he is with me since I know how to handle him*” (mother, rural). Consequently, caregivers reported that their concerns were never heard, “*even if we talk, who are we that they can listen to us*” (grandmother, rural). Caregivers feel inadequate to pursue office bearers to ensure that their children benefit from social services as provided in legally binding frameworks and policies. As such, caregivers’ call for enhancing caregiving entails the establishment of community spaces where rehabilitation programmes can be consistently conducted, especially when caregivers are trained.

### Theme 3: Empathy

Caregivers emphasized the role of “empathy”, rendered in the local language as “*Chisoni*”, helps them accept their child’s condition and cultivates both strength and resilience. During discussion sessions, we carefully distinguished “*Chisoni*” from “*Chifundo*”, a term meaning sympathy or pity. To enhance caregiving, empathy from family, community and professionals was perceived as a way for caregivers to strengthen caregiving efforts and openly allow their children to interact in community spaces.

#### Sub-theme: Empathy at family level.

At the helm of caregiving experiences is stigma, discrimination and exclusionary practices that begin in the nuclear family, with the majority of fathers being absent. To exemplify the ill perception of a child with a disability, caregivers shared and others validated “*the father cannot even drink from the same cup as the child, that is how bad it is*, *mine cannot even lift or hold the child*” (mother, urban). Another recollected, “*when I visit, they move their children away, I am given the most inhabitable space, maybe something that is supposed to be the kitchen, that’s where I have to sleep with my child even when they have a nice house and space, the reason they are doing this is because I am the one with a child that is sick*” (father, rural). An appalling 19 out of 25 caregivers reported no shared responsibility, the child belongs to the mother or maternal grandparents “*even when the child gets sick at night, it’s us as mothers who attend to them, fathers don’t agree to even hold the child, for them it a waste of time, energy and effort*” (mother, rural). Only 4 out of 23 female caregivers reported having journeyed with their spouses, equally contributing to child care. However, all caregivers lamented mistreatment from their husbands’ families, relatives and generally, community members. Families are difficult, “*for me, when our son was born, his paternal grandmother, arranged for him (my husband) to leave the home, and found a place for him to live, saying, in our family there is no such person with a disability, you should leave that home instead of being made to suffer*” (mother, urban). The lack of empathy in families resulted in caregivers being overprotective of themselves and their children. “*My wife died just after giving birth, ever since I have not remarried, and I will not because my job is taking care of him* (father, rural). Another recalled, “*for me, I would rather I keep my child for myself, even the time that I die, I will not know anything*” (mother, rural). Another said, “*for me, I would rather she dies first before me because there’s no one who can take care of her. In my family, some people are doing well but they cannot even come to visit me, they don’t even take care of normal children, what more mine who cannot even use the toilet*” (grandmother, rural). Such attitudes exacerbate care burden, “*there is no help, and relatives find excuses not to help at all costs*” (grandmother, urban). To make matters worse, this lack of consideration for the child with a disability extends to no visitations when the child is sick and hospitalized, “*our children frequently fall ill, yet people will not visit*” (mother, urban). Another narrated, “*for me, I have even told my family members that when I die, this child should be handed over to the government’s social welfare departme*nt *because I know that no relative of mine can keep this child* (father, rural).

#### Sub-theme: Empathy at community level.

Community anchors family, hospitals, schools, churches, marketplaces, shopping malls, play areas, and other spaces where caregivers and children interact. Caregivers recognise the cultural viewpoint of childcare in collectivistic settings like Zambia, which places the responsibility of raising children on the community. To exemplify this, Zambia’s collectivist culture on child-rearing practices typically embodies a communal obligation. This may take the form of neighbors escorting children to school, sharing meals in designated open spaces, and extended kin alternate in funding for childcare activities (e.g., clinic visits, medications, school fees). Such practices, however, rarely extend to children with disabilities. When a child has NDD for instance, there is significant decline in community members being part of the care process. There is clearcut exclusion from shared playgroups, shared meal times or a neighbor offering to feed the child. Additionally, relatives who would customarily rotate medical costs seldom contribute to repeated rehabilitation fees for a child with a disability. Thus, the communal safety net that protects children without disabilities largely unravels for families raising children with disabilities.

Community response to children with disabilities is most dreading, “*with my neighbours, when my child was born, he had scales like a crocodile, his eyes were looking like some sort of animal, and at eating time, he is given food to eat far away from everyone*” (mother, urban). These discriminatory tendencies get worse when expanded to extended family and community, “*when I attend funerals, I carry my child because I cannot leave him locked up at home, but when time to eat comes, these children eat with food falling out of their mouths, people will move away and leave you alone with your child, so you will look at yourself and say my child is not okay, so I carry the food and go and eat in a secluded place near a bush so that nobody has to see. When I finish feeding him, I clean him up and put him on my back*” (mother, rural). Caregivers experience a myriad of exclusionary practices, including alienation from family, community activities and unwholesome talks, which force them to withdraw and find themselves only in spaces where they are understood. With the declining physical and social spaces, caregivers report that their own lives are disrupted, lacking progress, feeling tied/trapped, held backwards because they cannot do what others are doing, experience care burnout because there is no one to share the care with and feel cheated and ill-fated. The non-existence of empathy in spaces where caregivers interacted was believed to have compromised caregiver, child functioning and well-being.

#### Sub-theme: Empathy at professional level.

In narrations, a child with a disability is primarily resident in the family and expands their horizon to include other contexts (i.e., hospitals and schools where they experience professionals). Equally, caregivers shared experiencing lack of empathy from professionals in clinics and hospitals. Due to lack of empathy, caregivers are despondent about seeking services from clinics whenever the child falls ill, but prefer to handle the illness at home. One narrated, “*some professionals are scared to touch our children, you cannot be close to them, you have to stand at a distance to be attended to*” (mother, rural). With consensus, caregivers recognized that attitudes when relating to caregivers and children is mostly negative, “*we are not treated like other people seeking services, it is not right, not encouraging at all, we are supposed to be running to them but look, most of us only go there only when the child is sick. We usually know that the child needs to be taken to the hospital, but you need to be mentally prepared to meet people who will cast you down further, it’s not easy*” (grandmother, rural). Another said, “*for me, my conclusion is that they do not have time to deal with our children, they appear to be in a hurry”* (mother, rural). Another narrated an experience where a physiotherapist exercised the child for only 5 minutes and, out of frustration, quit sessions when the child was 2 years old and started exercising him from home, “*see there he is, now he is walking*” (father, rural). We also observed an interesting view regarding the difficulties in interacting with a disability that one may never have experienced before, “mmmh……. *sometimes it can be scary but for us we do not have a choice, before now I had never experienced a child like this and I do not know if I could actually touch or lift a child, I do it because I do not have a choice*” (mother, rural). Regardless, and as the profession demands, caregivers concluded that, “*it is not our fault either that we have children with disabilities.* We *just found ourselves in this situation. We never thought that we would have a child with a disability, but here we are, and there is nothing we can do, and the fact remains, it can happen to anyone, and children with disabilities continue to be born, emphasising the obligation for clinics and people working there to actively engage and adapt in providing care and support”* (mother, rural).

### Theme 4: Thriving together

Caregivers clearly shared that having and caring for a child with an NDD was the most daunting, unimaginable, loneliest and difficult of tasks for anyone to experience. However, caregivers recognised how shared reality and mutual experiences created the base for resilience and thriving together in caregiving. Caregivers reported finding solace in community groups where sharing experiences of similar difficulties and successes was a treasured opportunity. These interactions were identified as the beacon of strength for caregivers, who were once directionless, largely isolated and absorbed in self-pity regarding the children’s life course. Caregiver lives were full of shame, sadness, loneliness, despair, stress, worry, and inadequacy. With an unsupportive social environment, experiences of solidarity and collective experiences made a difference for caregivers who expressed sentiments like, “*I thought I was alone*”, “*I have a place to belong where my child will be valued and treasured*,” (mother, rural), “*these conditions are common, it’s not witchcraft*” (grandmother, rural). To many caregivers, thriving together places responsibility on the support the community provides to collectively and proactively engage with one another to foster childcare. Thriving together bestows hope in the lives of caregivers as a ray of light amidst dark experiences. Through these community interactions, caregivers have experienced growth, personal development, and information, and through shared realities have become better representatives of their children. Caregivers can now speak openly about their children’s disabilities and extend open arms to those with new diagnoses. “*I am glad that I was connected to this group which is helping me to healthily deal with overwhelming family and community attitudes towards disability. I no longer feel alone, I have a community, and they have me. I offer what I was offered more so to newcomers because I know exactly how it feels to be isolated and forcibly put in a dark space by people you thought would be there no matter what*” (mother, urban). Another shared, “*I have learnt how to speak for my child. In my community, they used to shame my child, and I did not know what to do or say, I would cry and just leave them. But now everyone knows they cannot go free if they say mean words to my child. When I hear them, I follow them. Even when they are children, I go to talk to them and their parents. Through this group, I strongly advocate for my child, to an extent that people in my community are now seeing him like a human being*” (mother, rural). Caregivers thriving together has enriched and practically changed perception about disabilities and NDDs, “*it rebuilds your life. I have seen the way I just handle the people when they look at my child, even when they do not say anything. Previously, I would be hurt, but now I educate them because today it is me, next it will be another person in the community*” (mother, rural).

## Discussion

Overall, poor and restricted resources for early childhood development, evident in many LMICs, present a challenge for optimal developmental progression, particularly for children with more complex NDDs. Efficacy of early intervention is considered a significant precursor to developmental gains in NDDs, while emphasising the prominent contributions of primary caregivers [19,21,36,43,45,50]. In low-resourced settings, factors impeding early action for caregivers include poverty, absence of formal caregiving settings [31,38,68–70], lack of capacity, family support, accessible rehabilitative services, formal education, literacy and negative perception [8,18,19,22].

The insights from caregivers reveal some essential considerations required to optimise caregiving efforts using a family-centred approach. Our findings generally show challenges associated with NDDs caregiving, but also capacity needs that can facilitate caregiving. All caregivers were aware of their key role as caregiving agents for the children, recognising the significance of accurate NDDs information for effective care. All caregivers showed consensus on widespread and extensive knowledge of the development of typical children. This child’s knowledge is an inherent resourceful asset that was accumulated from childcare experiences even before having their children. On the contrary, the absence of information, knowledge and care skills for children with NDDs is “*loud*” [71], making caregiving a daunting experience on such a childcare “*detour*”. For caregivers, NDDs knowledge was fundamental for empowering childcare skills. In particular, NDDs knowledge on causes, treatments, supports and developmental pathways would inform not only “the why” but also the “how” of care. Without accurate information from the onset, caregivers were prone to experiment in the hope that the child would get better. A common experience across all caregivers was consulting alternative, traditional and religious treatments, which mainly focused on cleansing or casting out evil spirits tormenting the child. A key discomfort surrounding alternative treatments was the “*secrecy*” in practices. However, out of desperation, caregivers practised “*rituals*” with diligence in the hope that the child would be restored. Nonetheless, no child improvement was observed; instead, there was deterioration, especially in cases where ritual demands required withdrawal from conventional services. Additionally, caregivers realised that pivoting on witchcraft only increased hatred in families because family members were the ones identified as bewitching. Other studies conducted in traditional societies also reported similar findings of caregivers seeking alternate care systems [49], embedded in historical customs and beliefs to determine caregiving decisions and switching between treatment options [36,72–74]. Some researchers contend that families impacted by major health disruptions often find comfort in alternative treatments and expanded networks of support, including health and faith communities [75–77]

Although knowledge is reported to empower caregiver capacity [78] increase caregiver self-efficacy [79] and higher uptake of service usage [80], the absence of knowledge compromised caregivers’ care skills in this study. Investing in NDDs knowledge could be used to counter some of the deeply rooted cultural drivers that overwhelm and compel caregivers to action, as they search for quick solutions. This finding was not a surprise considering the low awareness levels of disability and poor efforts for social inclusion programmes. Currently, very few awareness efforts are driven by the government despite ratification and domestication of the United Nations Convention on the Rights of Persons with Disabilities [81], with an article emphasising awareness raising. Caregivers confirmed the low disability awareness, showcasing their own experience as an introduction to NDDs. This finding corroborates other findings of generally low awareness and knowledge levels of developmental conditions in poor countries [20,82–84] and their effects on child care [5,85]. Szlamka and colleagues [20] reported similar low uptake of awareness programmes by governments, reporting only pockets of initiatives led by affected parents. Another explanation for continued records of low NDDs knowledge could relate to high illiteracy levels among caregivers. Some caregivers acknowledged undergoing disability training several times but experienced challenges internalising information due to illiteracy and difficulties understanding disability concepts in the English language. Other studies sampling rural and poor communities report similar education and literacy challenges [18,19,22] in understanding NDDs. A way to enhance knowledge, learning and retention would be through psychoeducation and using simple, easy-to-read language. Information retention is significant for cascading information to family members who complement caregiving efforts. Another knowledge and information lag, described as shockingly unexpected, was among the healthcare professionals and clinicians. Despite placing “*clinicians*”, especially doctors, in high esteem, caregivers received little helpful information to support child care skills beyond diagnosis. The limited professional knowledge on NDDs plunged caregivers into a panic, desperate, vulnerable state as though fighting a losing battle. Information obtained from clinicians was related to caregivers exercising “*patience*” in care, considering that the child would be slow, not develop like other children, without sufficient explanation of why the child turned out that way. These findings confirm similar trends in many LMICs where there is a paucity of NDDs knowledge among professionals [1,83,84,86]. This study’s findings provide an opportunity for addressing the NDDs knowledge gap for health professionals, necessary for understanding and application of care skills for child support.

After knowledge, caregivers identified rehabilitation services as essential to caregiving. All the children in the study required various rehabilitation services, including medical, physiotherapy, educational, occupational, speech and psychological therapies, in order to increase functionality. Particularly for Zambia, research and anecdotal evidence show pressing rehabilitation needs for children with disabilities, but access remains far-fetched [1]. While caregivers acknowledge the pivotal role of rehabilitation services for increasing child gains and easing caregiving, these services are limited to medical facilities. We found caregiver rehabilitation services knowledge to be limited to assistive devices—wheelchairs, standing frames or adapted chairs and physiotherapy. Other services like occupational, speech, social, psychological and educational services were not apparent, largely because the services are non-existent. To address access to rehabilitation services, recognising ways that restrict current access is paramount. We mapped caregivers’ descriptions of experiences with rehabilitation services onto the five key elements proposed by Penchansky and Thomas [87]. First, caregivers reported accessibility difficulties characterised by poor physical connection between the family and the location of rehabilitation services. As a result, children inconsistently attended services due to transportation costs, and others with assistive devices like wheelchairs could hardly use them due to the poor state of the roads and general terrain in communities. Second, caregivers report poor acceptability, showing overall low satisfaction with health care services. For example, physiotherapy services were not only inadequate but there was reluctance, resistance and ambivalence among health professionals to work with the children. With health practitioners displaying a negative attitude or fear of working with the children, some caregivers quit attending facility services and resorted to conducting their sessions at home. Third, the affordability of services was out of reach for caregivers due to obvious financial constraints and non-existent health insurance to support the uptake of services. Fourth, the availability of rehabilitation services does not, at the moment, meet community demand. Lastly, appropriateness of rehabilitation services ensures that services, goals, and assistive devices match the individual child’s needs. These findings were expected considering poor provision of medical rehabilitation services hindered by poor budget allocation, lack of human resource capacities and inaccessible health and social services [8,88–90]. Our findings suggest that caregivers require more than what is currently on offer to enhance caregiving efforts through accessing full rehabilitation services as demanded by the child’s needs. In addition to sensitising caregivers on full rehabilitation services beyond those targeting mobility, opening community spaces where caregivers can access not only training but also community workspaces within their communities as a beneficial strategic direction. Creating these community spaces will produce threefold benefits: 1) increase disability awareness; 2) increase rehabilitation dose, and 3) caregiver solidarity.

The other essential ingredient for enhancing caregiving skills was “*empathy*”. Caregivers who reported experiencing empathy from immediate family members described having a deep sense of strength and resilience. However, only four (4) caregivers shared receiving unwavering support from spouses, and at the same time, shielding them from unwholesome talks from extended families. Most caregivers described experiencing estrangement across the different levels of proximal interactions. Our findings show that lack of empathy fuels caregiver emotions regardless of child age or functionality. Sharing these experiences during sessions triggered intense emotions, including crying. Descriptions of personal, emotional triggering experiences were not easy for caregivers to share, but they gathered courage to do so because the group was safe and compassionate. Beneath the blanket statement “*we go through a lot*” were many horror stories where children were denied, wished dead, mothers blamed, and marriages broken down. From our analysis, caregivers experiencing exclusionary practices were a result of disability intolerance in families and communities. Other studies report experiences of disregard and disengagement by family, friends and medical professionals as fuel for social isolation and exclusion for caregivers [91]. To a larger extent, caregivers painfully experienced a dwindled social and physical world characterised by alienation, hostility and segregation, compelling them “*to keep*” (hide) their children away from the public. Most caregivers were treated as outcasts, kept at arm’s length, and rarely invited to participate in significant community activities like funerals and weddings. Beyond family and community, these repulsive experiences extended to clinics and hospitals. Despite being perceived as “*empathy centres*”, caregivers experienced a fair share of stigma and discriminatory attitudes in health facilities. Empathy was dependent on one’s luck or professionals’ mood, but generally, the demeanour, unwelcoming attitude, and negative emotional attunement of professionals were not inspiring for hospital visits, explaining the infrequent visits, missing reviews and scheduled appointments. Notwithstanding the many counterproductive experiences, caregivers exhibited a cohesive, thriving-together attitude that reflected collective experiences. This togetherness was strengthened and exemplified by shared reality, common purpose, and exchanging practical ideas for thriving together. These groups have recreated and elevated the sense of belonging and togetherness that was absent in communities. Caregivers have been empowered to reach out to newcomers, offering support and a platform using practice-based evidence. In efforts of togetherness and solidarity, caregivers proactively look out for one another, driven by the philosophy that NDDs knowledge, access to community rehabilitation spaces, caregiver wellbeing (empathy), determines caregiving efforts, child developmental outcomes and wellbeing.

## Implications and conclusions

The high stakes associated with delayed intervention for children with developmental disabilities cannot be overemphasised. Aside from the contextual difficulties of poor resources experienced in many LMICs, following a family-centred approach to early intervention is a promising direction. Clearly, interactions between health facilities and families remain critical for the advancement of early identification and intervention. To counter the paucity of NDDs knowledge among professionals and caregivers, introducing NDDs content in health training institutions and in curricula for antenatal classes could improve the application of early services and intervention decisions. In similar ways, medical professionals could benefit from continuous professional development programmes on empathy and disability inclusion. Discussions with caregivers highlighted the following takeaways for the caregivers: 1) immature or faulty development of the brain cause NDDs; 2) the role of early intervention on brain development and skill acquisition; 3) proactive engagement of caregivers in management of NDDs, taking advantage of the young brain. Investing in increasing disability knowledge in general and NDDs in particular is an indisputable way of increasing disability tolerance and empathy while countering exclusionary practices. Caregivers, through their own initiatives are already raising disability awareness as they await government efforts. Access to rehabilitative services and education is the right of every child; therefore, ensuring the presence of quality services in local communities increases uptake of services and eventual developmental gains for children with NDDs.
